# Body mass index but not genetic risk is longitudinally associated with altered structural brain parameters

**DOI:** 10.1038/s41598-021-03343-3

**Published:** 2021-12-20

**Authors:** Anne Tüngler, Sandra Van der Auwera, Katharina Wittfeld, Stefan Frenzel, Jan Terock, Nele Röder, Georg Homuth, Henry Völzke, Robin Bülow, Hans Jörgen Grabe, Deborah Janowitz

**Affiliations:** 1 Department of Psychiatry and Psychotherapy, University Medicine Greifswald, Ellernholzstraße 1-2, 17475 Greifswald, Germany; 2 German Center for Neurodegenerative Diseases DZNE, Site Rostock/Greifswald, Ellernholzstraße 1-2, 17475 Greifswald, Germany; 3 Department of Psychiatry and Psychotherapy, HELIOS Hanseklinikum Stralsund, Rostocker Chaussee 70, 18437 Stralsund, Germany; 4 Interfaculty Institute for Genetics and Functional Genomics, University Medicine Greifswald, Felix-Hausdorff-Str. 8, 17475 Greifswald, Germany; 5 Institute for Community Medicine, University Medicine Greifswald, Walther-Rathenau-Str. 48, 17487 Greifswald, Germany; 6 Department of Diagnostic Radiology and Neuroradiology, University Medicine Greifswald, Ferdinand-Sauerbruch-Str., 17475 Greifswald, Germany

**Keywords:** Brain, Risk factors, Epidemiology

## Abstract

Evidence from previous studies suggests that elevated body mass index (BMI) and genetic risk for obesity is associated with reduced brain volume, particularly in areas of reward-related cognition, e.g. the medial prefrontal cortex (AC-MPFC), the orbitofrontal cortex (OFC), the striatum and the thalamus. However, only few studies examined the interplay between these factors in a joint approach. Moreover, previous findings are based on cross-sectional data. We investigated the longitudinal relationship between increased BMI, brain structural magnetic resonance imaging (MRI) parameters and genetic risk scores in a cohort of n = 502 community-dwelling participants from the Study of Health in Pomerania (SHIP) with a mean follow-up-time of 4.9 years. We found that (1) increased BMI values at baseline were associated with decreased brain parameters at follow-up. These effects were particularly pronounced for the OFC and AC-MPFC. (2) The genetic predisposition for BMI had no effect on brain parameters at baseline or follow-up. (3) The interaction between the genetic score for BMI and brain parameters had no effect on BMI at baseline. Finding a significant impact of overweight, but not genetic predisposition for obesity on altered brain structure suggests that metabolic mechanisms may underlie the relationship between obesity and altered brain structure.

## Introduction

Overweight (BMI = body mass index, kg/m^2^, BMI > 25 kg/m^2 1^) and obesity (BMI > 30 kg/m^2^^1^) represent serious challenges for international health care systems. Since 1975, the number of people with obesity has more than tripled^1^ with now 1.9 billion adults worldwide affected and 650 million of those being obese^1^. Factors causing obesity and overweight are manifold and not yet well understood. In addition to behavioral factors like unfavorable diet and low physical exercise, converging evidence points at a significant influence of genetic predisposition promoting the development of obesity^2^.

In their large-scale genome-wide association study (GWAS), Locke et al. (2015) identified 97 BMI-associated loci^3^. Moreover, many of these genetic variants were found to be enriched particularly in the central nervous system, suggesting that genes involved in weight regulation and obesity may directly impact on obesity-related structural brain changes. A more recent meta-analyses with N = 700,000 probands was able to confirm and reinforce these results^4^.

In addition to the association of obesity with diverse health conditions and particularly cardiovascular diseases^5^, converging evidence suggests that overweight and obesity are related to brain structural and functional impairments. For example, a previous study of our working group found highly significant associations between abdominal obesity and reduced gray matter volumes^6^ (GMV). Likewise, in a cross-sectional study using data from the UK Biobank, Hamer and Batty found that both, BMI and waist-to-hip-ratios were important predictors of brain gray matter atrophy^7^. Another, very recent, large-scale UK Biobank study by Gurholt et al. also showed negative associations between specific brain areas and anthropometric factors such as BMI, waist-to-hip-ratio and waist circumference^8^.

Results on brain regions which are primarily affected by obesity remained heterogeneous to some degree: For example, findings by Pannacciulli et al. illustrated that high BMI values are associated with reduced GMV in impulse control and reward-related brain areas including the post-central gyrus, frontal operculum, putamen and middle frontal gyrus^9^. Some studies even showed that high BMI levels are associated with hemispheric asymmetries in obesity^10,11^. Alonso-Alonso et al. found that elevated BMI levels are associated with low values in cortical thickness in right frontal lobe and high values in thickness in the left frontal lobe^11^.

A recent meta-analysis by Chen et al. study showed BMI-associated decrease in GMV in the orbitofrontal cortex (OFC), a region which also showed strong responses to visual and olfactory food cues^12^. Still, findings of another meta-analyses by Garcia-Garcia et al. emphasized the involvement of the medial prefrontal cortex (mPFC)^13^ in the relation between increased BMI and reduced gray matter volumes.

Applying an integrative approach, Opel et al. combined (2017) the genetic basis of high BMI, obesity and gray matter alterations using a cross-sectional design^14^. The authors found evidence for associations between the genetic risk for increased BMI levels and reduced gray matter volumes suggesting that gray matter alterations represent an unavoidable transitional phase in the development of pronounced obesity. In a more recent study of this working group, the authors could replicate their major findings of associations between overweight and obesity on one hand and cortical as well as subcortical abnormalities on the other hand^15^. In addition, the polygenic risk score for obesity was directly correlated with reduced occipital surface area.

Another source of heterogeneity in existing findings is the use of different parameters of brain structure, i.e. cortical thickness, surface area and cortical volumes. Evidence showed that these outcomes are genetically and phenotypically independently determined^16^. Moreover, most previous studies on the relationship between overweight/ obesity, the corresponding genetic variants and brain structural alterations were based on cross-sectional data, which limits their explanatory power.

To further examine the association between brain alterations, BMI-related genes and obesity, investigations using longitudinal designs are needed. This approach appears particularly important in the light of findings suggesting that obesity may represent as an accelerator for aging processes in the brain^17^.

To overcome these shortcomings, we examined the effects of increased BMI values on various structural brain parameters—cortical thickness, volume, surface area—analyzed these parameters in two regions of interest—the orbitofrontal cortex (OFC) and the anterior cingulate and medial prefrontal cortex (AC-PFC)—areas which were recently identified in meta-analytic studies^12,13^, are functionally closely interconnected and which are linked to eating behavior, and assessed them twice across a time period 4.9 years on average in a large and well-characterized general-population sample. Specifically, we sought to determine whether increased BMI values can predict future reductions in structural brain parameters and vice versa. Additionally, we investigated genetic risk as a possible mechanism for increased BMI and reduced GMV both in a longitudinal and cross-sectional design.

## Materials and methods

### Study population

Data from the Study of Health in Pomerania (SHIP) were used^18^. The target population was comprised of adult German residents in northeast Germany living in three cities and 29 communities, with a total population of 212,157. A two-stage stratified cluster sample of adults aged 20–81 years had been drawn from local population registration files. The net sample comprised 6267 eligible participants, of which 4308 Caucasian individuals participated at baseline SHIP-0 between 1997 and 2001. In 2008, the 11-year-follow-up (SHIP-2) of the population-based study started, examining 2333 individuals. From 2014 to 2016 the third follow-up examination (SHIP-3, 17-year-follow-up) was carried out (n = 1718). All participants from SHIP-0 were invited back for both follow-ups and underwent basic medical examinations.

Additionally, participants from SHIP-2 and SHIP-3 were asked to participate in a whole-body MRI assessment^19^. In total, 1163 individuals from SHIP-2 and 868 individuals from SHIP-3 underwent the MRI. Thus, approximately 50% of all individuals were eligible for the MRI study and volunteered to participate. A complete description of the study was provided to the participants, a written informed consent was obtained.

### Interview and clinical examination

Medical history and sociodemographic factors were assessed in a standardized questionnaire by means of a computer-assisted face-to-face interview. Body measurements such as height and weight were measured by staff and later used to calculate the BMI (kg/m^2^).

### MRI assessment

Longitudinal MRI data was available for n = 686 individuals. After exclusion of medical conditions (e.g., history of cerebral tumor, stroke, Parkinson's disease, epilepsy) or poor technical quality (e.g., severe movement artefacts), longitudinal MRI data was available for n = 607 participants in SHIP-2 and SHIP-3. After applying quality control measures^18^, including a plausibility check, and then excluding a number of participants, the final study sample was n = 502.

All images were obtained using a 1.5 T scanner (Magnetom Avanto, Siemens Healthineers, Erlangen, Germany) with a T1-weighted magnetization prepared rapid acquisition gradient echo (MPRAGE) sequence. Anatomic T1-weighted images were acquired using a three-dimensional axial, multiplanar reconstruction MRI with the following sequence parameter: 1900 ms repetition time, 3.4 ms echo time, flip angle = 15° and a voxel size of 1.0 × 1.0 × 1.0 mm^3^. The duration of the MRI brain protocol performed in this study was 3:38 min^20^.

For further information on MRI segmentation, used brain atlas specifications and description of statistical outlier control, see “MR image segmentation”.

### MR image segmentation

Cortical reconstruction was performed with the FreeSurfer image analysis suite (FreeSurfer Version 6, 2017), which is documented and freely available for download at: http://surfer.nmr.mgh.harvard.edu.

Briefly, this processing includes removal of non-brain tissue using a hybrid watershed/surface deformation procedure^21^ automated Talairach transformation, intensity normalization^22^, tessellation of the gray matter white matter boundary, automated topology correction^23,24^ and surface deformation following intensity gradients to optimally place the gray/white and gray/cerebrospinal fluid borders at the location where the greatest shift in intensity defines the transition to the other tissue class^25–27^.

Once the cortical models are complete, individual images are being registered to a spherical atlas which is based on individual cortical folding patterns to match cortical geometry across participants^25^. FreeSurfer also gives an estimate of the total intracranial volume (ICV) based on linear transformations to MNI305 space^21^.

In order to accurately define the boundaries of the target regions we projected a recently published cortical atlas by Glasser et al. to the template used for spherical registration by FreeSurfer^28^. Using this projection new annotations of the cortical models of all individuals and time points were computed.

### Prefrontal cortex regions

The atlas proposed by Glasser et al. comprises 180 regions per hemisphere which were delineated using cytoarchitecture, task-related fMRI, and functional connectivity. Previous brain maps only used one neurobiological property to describe brain areas. Glasser et al. combined all properties, so a more wholesome approach was possible. Therefore, in this study, the two target regions were defined based on this atlas. The first one is the orbital and polar part of the frontal cortex (OPFC), further referred to as orbitofrontal cortex OFC (see Supplement 1). The orbitofrontal cortex is believed to be part of a larger functional network which includes the orbitofrontal cortex, the medial prefrontal cortex, and the anterior cingulate cortex^29^. Therefore, we also considered the anterior cingulate and medial prefrontal cortex (AC-MPFC) (see Supplement 1). For both target regions mean cortical thicknesses, volumes, and surface areas were computed. Left and right hemisphere for region of interest (ROI) analyses was conducted separately. In addition, we also considered mean cortical thickness, volume, and surface area of the whole left and right hemisphere.

Quality control of all volumetric measures was implemented by exclusion of cases which were more than three standard deviations away from the whole sample mean after adjusting for age, sex, and intracranial volume (N = 10). Variables for mean cortical thickness, volume and surface area of the OFC and AC-MPFC, left and right hemisphere (left and right hemisphere separately in SHIP2 and SHIP-3), were calculated as follows. Volume and surface area of OFC and AC-MPFC were defined as the sum of the volume or area of the included structures. Mean cortical thickness was calculated as an area weighted sum according to the included structures.

$$\mathop \sum \nolimits_{{i = 1}}^{n} \frac{{ area\left( i \right)}}{{area\left( {total} \right)}}*thickness\left( i \right) $$

In an additional statistical quality control, we excluded participants with values outside a three standard deviation boundary in each of the N = 24 parameters leaving a final complete data set for analyses of N = 502 participants.

### Genetic data

SHIP participants were genotyped using Human SNP Array 6.0 (Affymetrix, Santa Clara, California, SNP = Single Nucleotide Polymorphism). The overall genotyping efficiency was 98.55%. Imputation of genotypes in the SHIP cohort was performed with the software IMPUTE v2.2.2 against the 1000 Genomes (v1.3, Build 37) reference panel using 869,224 genotyped SNPs. For details on imputation and quality control (QC) for the SHIP sample see Teumer et al.^30^. The genetic profile scoring was based on the summary results from the meta-analysis on BMI by Locke et al.^3^. SNP information including *p* values and betas were obtained by using PLINK^31^.

To identify polygenic effects owing to independent SNPs in linkage equilibrium, SNPs were pruned based on variance inflation and a pairwise *R*^2^ threshold of 0.1 and a sliding window of 50 SNPs shifting 5 SNPs at each step. We excluded SNPs on chromosomes X and Y, mitochondrial SNPs, and SNPs with a minor allele frequency < 0.01, genotype missing rate > 0.05, deviation from Hardy–Weinberg equilibrium (*p* < 0.001). The polygenic risk score (PRS) for each subject were calculated only based on SNPs with a p-value *p* ≤ 0.05 in the BMI GWAS to catch only BMI relevant signals and to avoid multiple testing. For each SNP, the number of risk variants (0–2) in individual carriers was multiplied by the beta estimate for the particular variant from the GWAS results.

### Statistical analyses

Descriptive information on the variables was given as means and standard deviations for metric variables. Group differences between SHIP-2 and SHIP-3 were tested for statistical significance with T-tests (continuous data). A value of p < 0.05 was considered statistically significant.

Multiple linear regression models were performed to assess the association between brain structures, BMI and genetic predisposition for BMI (source code information available in supplement material).

Different hypotheses were tested.

0. B Baseline cross-sectional analysis: BMI at SHIP-2 is associated with brain variables in SHIP-2: For each of the 18 brain outcomes at SHIP-2 (Brain_S2) a linear regression model with robust estimates was fitted adjusted for age at the time point SHIP-2 (age_S2), sex, sex*age interaction term and intracranial volume at SHIP-2 (icv_S2). Predictor of interest was BMI at SHIP-2 (BMI_S2).
  $$ {\text{Brain}}\_{\text{S2}}\sim {\text{BMI}}\_{\text{S2}} + {\text{icv}}\_{\text{S2}} + {\text{age}}\_{\text{S2}} + {\text{sex}} + {\text{age}}\_{\text{S2}}*{\text{sex}} $$
1. BMI at SHIP-2 (baseline) predicts brain atrophy in SHIP-3 (follow-up): For each of the 18 brain outcomes at SHIP-3 (Brain_S3) a linear regression model with robust estimates was fitted adjusted for age at the time point of SHIP-2 (age_S2), sex, age*sex interaction term, follow up time between SHIP-2 and SHIP-3 (fup_time), brain parameter at SHIP-2 (Brain_S2) and intracranial volume at SHIP-2 (icv_S2). Predictor of interest was BMI at SHIP-2 (BMI_S2).
  $$ {\text{Brain}}\_{\text{S3}}\sim {\text{BMI}}\_{\text{S2}} + {\text{Brain}}\_{\text{S2}} + {\text{icv}}\_{\text{S2}} + {\text{age}}\_{\text{S2}} + {\text{sex}} + {\text{age}}\_{\text{S2}}*{\text{sex}} + {\text{fup}}\_{\text{time}} $$
2. Brain parameters at SHIP-2 predict BMI at SHIP-3: A linear regression model for BMI was fitted adjusted for the same covariates as in 1. Predictors of interest were the 18 brain variables in SHIP-2.
  $$ {\text{BMI}}\_{\text{S3}}\sim {\text{Brain}}\_{\text{S2}} + {\text{BMI}}\_{\text{S2}} + {\text{icv}}\_{\text{S2}} + {\text{age}}\_{\text{S2}} + {\text{sex}} + {\text{age}}\_{\text{S2}}*{\text{sex}} + {\text{fup}}\_{\text{time}} $$
3. The genetic score for BMI predicts brain atrophy in SHIP-3: For each of the 18 brain parameters linear regression models were fitted adjusted for the same covariates as in 1, including the (PRS) for BMI as predictor of interest. Analyses were calculated with and without additional adjustment for BMI.
  $$ {\text{Brain}}\_{\text{S3}}\sim {\text{PRS}} + {\text{Brain}}\_{\text{S2}} + {\text{icv}}\_{\text{S2}} + {\text{age}}\_{\text{S2}} + {\text{sex}} + {\text{age}}\_{\text{S2}}*{\text{sex}} + {\text{fup}}\_{\text{time }}\left( { + {\text{BMI}}\_{\text{S2}}} \right) $$
4. Combined effect of an interaction of brain parameters and the genetic score for BMI on measured BMI: A linear regression model for BMI in SHIP-2 was fitted adjusted for age_s2, sex, their interaction and follow up time. Predictor of interest was the interaction term between the 18 brain models in SHIP-2 and the genetic score for BMI.
  $$ {\text{BMI}}\_{\text{S2}}\sim {\text{Brain}}\_{\text{S2}}*{\text{PRS}} + {\text{Brain}}\_{\text{S2}} + {\text{PRS}} + {\text{icv}}\_{\text{S2}} + {\text{age}}\_{\text{S2}} + {\text{sex}} + {\text{age}}\_{\text{S2}}*{\text{sex}} + {\text{fup}}\_{\text{time}} $$

In all analyses age was treated nonlinear as restricted cubic splines with four knots based on Harrell et al., 2001^32^. In all multiple regression models robust variance estimates were used. All analyses were performed with STATA 14.

We aimed to reduce the number of tests by combining the given information into whole hemisphere parameters (see “Statistical analyses”). Multiple regression models for whole brain, OFC and AC-MPFC (left, right; cortical thickness, volume, surface area) were carried out resulting in n = 18 models. Our main focus was on cortical thickness with surface area and volume as additional analyses. As the values for both hemispheres were highly correlated (r between 0.84 and 0.99, Table 3) we corrected for n = 3 tests in each sub-analysis (1)—(4), one for each anatomic structure (whole brain, OFC and AC-MPFC), respectively (p_corrected_ = 0.017).

### Ethics approval

The study was carried out in accordance with the Declaration of Helsinki, including written informed consent of all participants included in the study. The survey and study methods of both the studies were approved by the institutional review boards of the University of Greifswald.

## Results

Table 1 summarizes the clinical characteristics of the study participants in the baseline (SHIP-2) and follow-up (SHIP-3) samples with available longitudinal brain MRI data at times of measurement. Participants differed in age and showed significant atrophy in the global brain MRI characteristics in SHIP-3 as compared to SHIP-2. There were no statistically significant differences in BMI between SHIP-2 and SHIP-3.

**Figure 1:**
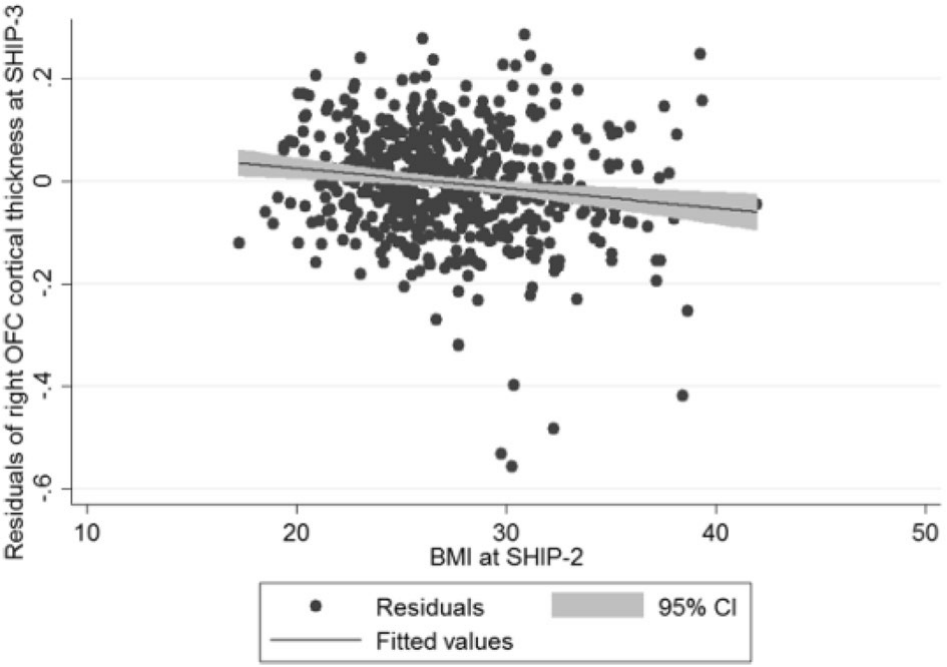
Scatter plot of BMI measured at SHIP-2 against the adjusted residuals for right OFC cortical thickness at SHIP-3 (adjusted for right OFC cortical thickness at SHIP-2, age, sex, age*sex interaction, ICV, follow-up time).

**Figure 2:**
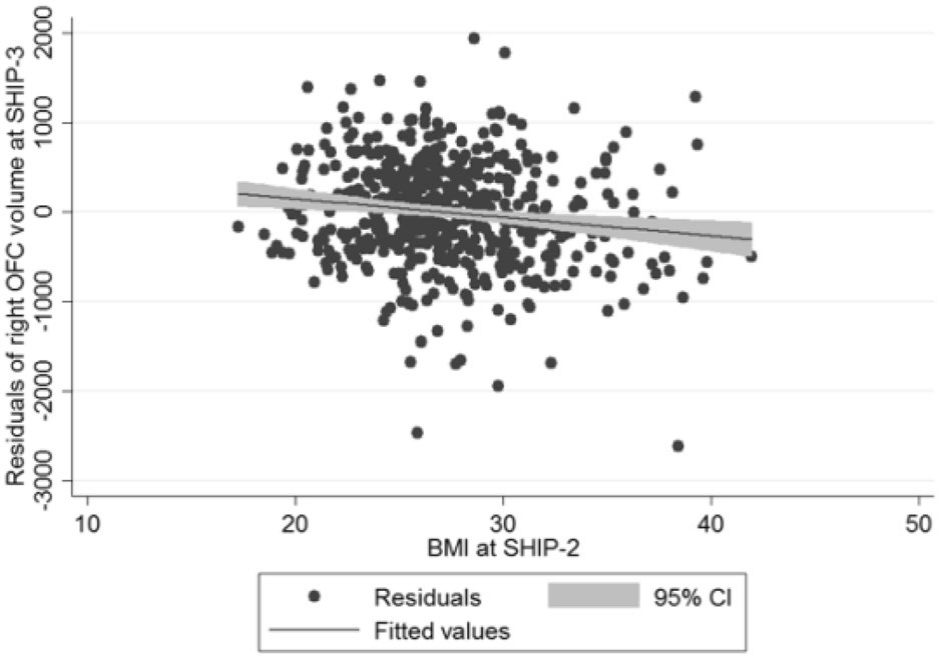
Scatter plot of BMI measured at SHIP-2 against the adjusted residuals for right OFC volume at SHIP-3 (adjusted for right OFC volume at SHIP-2, age, sex, age*sex interaction, ICV, follow-up time).

**Figure 3:**
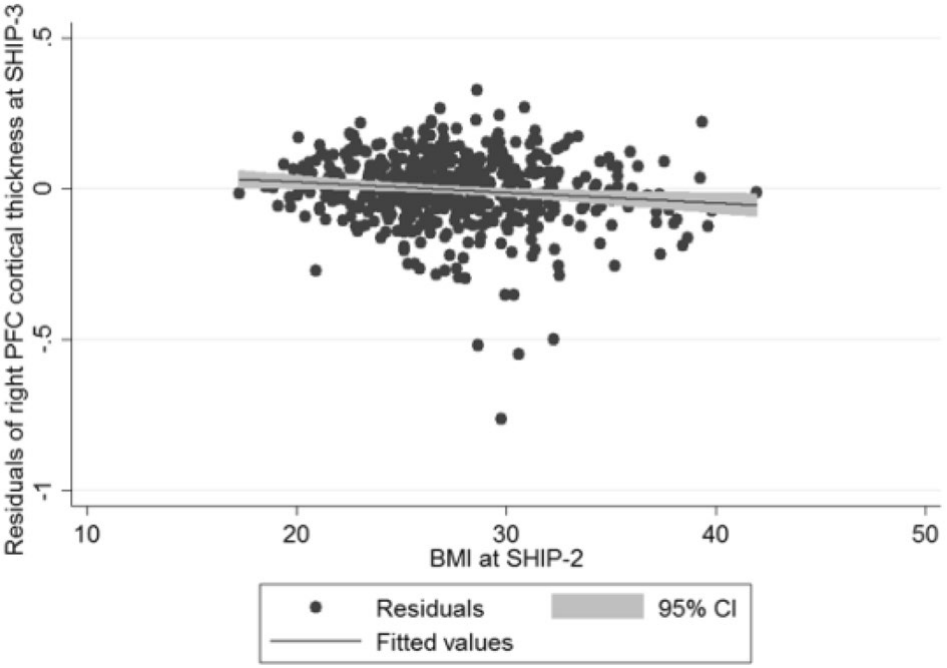
Scatter plot of BMI measured at SHIP-2 against the adjusted residuals for right AC-MPFC mean cortical thickness at SHIP-3 (adjusted for right AC-MPFC mean cortical thickness at SHIP-2, age, sex, age*sex interaction, ICV, follow-up time).

0. First, we tested the cross-sectional effect of BMI on brain parameters in SHIP-2. Especially for the prefrontal cortex the BMI showed a strong negative association (see supplementary Table S).
1. After this, we evaluated the effect of BMI on brain parameters in a longitudinal setting (see Table 1). BMI revealed no significant effect on whole brain gray matter parameters. Regarding our regions of interest (OFC and AC-MPFC) BMI at SHIP-2 was associated with reduced mean cortical thickness and GMV of the OFC and reduced mean cortical thickness of the AC-MPFC. These effects were found for both hemispheres in the OFC and for the right hemisphere in the AC-MPFC (see Table 2 and Figs. 1, 2 and 3). Brain surface area at SHIP-3 was not significantly associated with BMI at SHIP-2.
2. In a next step we tested the effect of baseline brain parameters on follow-up BMI. No brain parameter in SHIP-2 revealed a significant effect on BMI in SHIP-3 (see Table 3).
3. Thirdly, we evaluated the effect of the PRS for BMI on longitudinal brain changes in SHIP. The PRS for BMI revealed no significant effect on the tested brain structures irrespective of the additional adjustment for BMI (see Table 4).
4. In a last step, we evaluated the cross-sectional interaction between the PRS for BMI and brain parameters on measured BMI in SHIP-2. No significant interaction between any brain parameter and PRS for BMI on measured BMI in SHIP-2 could be observed (see Table 5).

**Table 1:** Description of SHIP-2 and SHIP-3 sample with available longitudinal MRI data (N = 502).

Mean (sd)	SHIP-2	SHIP-3	Comparison
Age in years	55.3 (12.0) Range: 31–82	60.2 (12.0) Range: 36–87	T = −140, P < 0.001
Men/women	221/281	221/281	
BMI	27.4 (4.2)	27.6 (4.5)	T = −1.8, P = 0.08
Mean cortical thickness^a^ (mm)	2.344 (0.11)	2.323 (0.12)	T = 10.7, P < 0.001
Mean volume^a^ (mm^3^)	225,323 (23,169)	221,099 (23,172)	T = 23.8, P < 0.001
Mean area^a^ (mm^2^)	87,164 (8675)	86,274 (8588)	T = 19.5, P < 0.001
Intracranial volume (mm^3^)	1,565,015 (160,573)	1,565,015 (160,573)	P = 1

^a^Mean of left and right hemisphere, mean volume refers to mean GMV.

**Table 2:** Results for longitudinal effects of BMI at SHIP-2 on brain parameters at SHIP-3.

Outcome	Coefficient	P-value	95% confidence interval
**Whole brain**			
Right cortical thickness	β = −0.0009	0.078	[−0.002, 0.0001]
Left cortical thickness	β = −0.0008	0.094	[−0.002, 0.0001]
Right volume	β = −66.04	0.12	[−148.42, 16.33]
Left volume	β = −49.63	0.25	[−133.89, 34.63]
Right surface area	β = 18.65	0.14	[−6.31, 43.62]
Left surface area	β = 29.45	0.026	[3.47, 55.43]
**Orbitofrontal cortex**			
Right cortical thickness	**β = −0.0037**	**0.0055**	**[−0.006, −0.001]**
Left cortical thickness	**β = −0.0034**	**0.006**	**[−0.006, −0.001]**
Right volume	**β = −20.80**	**0.0032**	**[−34.61, −6.99]**
Left volume	**β = −20.33**	**0.0032**	**[−33.80, −6.86]**
Right surface area	β = 2.53	0.29	[−2.12, 7.19]
Left surface area	β = 1.48	0.51	[−2.90, 5.86]
**Anterior cingulate and medial prefrontal cortex**			
Right cortical thickness	**β = −0.003**	**0.0045**	**[−0.006, −0.001]**
Left cortical thickness	β = −0.003	0.037	[−0.005, −0.0002]
Right volume	β = −10.70	0.077	[−22.58, 1.18]
Left volume	β = −4.80	0.39	[−15.86, 6.27]
Right surface area	β = 1.09	0.55	[−2.53, 4.71]
Left surface area	β = 1.97	0.24	[−1.35, 5.30]

Significant results are highlighted in bold (p<0.017); analyses are adjusted for ICV, age_2, sex, age*sex interaction, brain parameter at SHIP2 and follow-up time, volume refers to GMV.

**Table 3:** Results for longitudinal effects of brain parameters at SHIP-2 on BMI at SHIP-3.

Predictor	Coefficient	P-value	95% confidence interval
**Whole brain**			
Right cortical thickness	β = 0.02	0.98	[−1.34, 1.37]
Left cortical thickness	β = 0.17	0.80	[−1.19, 1.53]
Right volume	β = 7.94E−6	0.21	[−4.6E−6, 0.00002]
Left volume	β = 8.3E−6	0.22	[−4.9E−6, 0.00002]
Right surface area	β = 0.00002	0.30	[−0.00002, 0.00005]
Left surface area	β = 0.00001	0.43	[−0.00002, 0.00005]
**Orbifrontal cortex**			
Right cortical thickness	β = 0.03	0.94	[−0.76, 0.82]
Left cortical thickness	β = −0.09	0.83	[−0.96, 0.77]
Right volume	β = 0.00007	0.24	[−0.00005, 0.0002]
Left volume	β = 0.0001	0.17	[−0.00004, 0.0002]
Right surface area	β = 0.0001	0.47	[−0.0002, 0.0005]
Left surface area	β = 0.0003	0.15	[−0.0001, 0.0007]
**Anterior cingulate and medial prefrontal cortex**			
Right cortical thickness	β = 0.36	0.42	[−0.52, 1.25]
Left cortical thickness	β = 0.33	0.47	[−0.57, 1.24]
Right volume	β = 0.00009	0.18	[−0.00004, 0.0002]
Left volume	β = 0.0001	0.36	[8.9E−6, 0.0003]
Right surface area	β = 0.00009	0.64	[−0.0003, 0.0005]
Left surface area	β = 0.0002	0.34	[−0.0002, 0.0006]

Analyses are adjusted for ICV, age_2, sex, age*sex interaction, BMI at SHIP-2 and follow-up time, volume refers to GMV.

**Table 4:** Results for longitudinal effects of the genetic score for BMI on brain parameters at SHIP-3 with and without additional adjustment for BMI.

Outcome	Without adjustment for BMI	With adjustment for BMI
	Effect (pos/neg), p-value	Effect (pos/neg), p-value
**Whole brain**		
Right cortical thickness	β = −1.57, p = 0.16	β = −1.20, p = 0.3
Left cortical thickness	β = −0.74, p = 0.52	β = −0.32, p = 0.78
Right volume	β = −107,878, p = 0.28	β = −80,533, p = 0.44
Left volume	β = −54,111, p = 0.61	β = −31,855, p = 0.76
Right surface area	β = 15,043, p = 0.55	β = 5177, p = 0.84
Left surface area	β = 969, p = 0.97	β = −16,399, p = 0.55
**Orbifrontal cortex**		
Right cortical thickness	β = −0.12, p = 0.96	β = 2.0, p = 0.46
Left cortical thickness	β = −0.40, p = 0.87	β = 1.66, p = 0.52
Right volume	β = 3875, p = 0.81	β = 16,019, p = 0.32
Left volume	β = −4505, p = 0.74	β = 7428, p = 0.60
Right surface area	β = −2083, p = 0.70	β = −3508, p = 0.54
Left surface area	β = −5331, p = 0.34	β = −6571, p = 0.26
**Anterior cingulate and medial prefrontal cortex**		
Right cortical thickness	β = −0.43, p = 0.89	β = 1.46, p = 0.63
Left cortical thickness	β = −1.68, p = 0.51	β = −0.26, p = 0.92
Right volume	β = 16,487, p = 0.25	β = 24,163, p = 0.098
Left volume	β = −4483, p = 0.72	β = 2087, p = 0.87
Right surface area	β = 6428, p = 0.17	β = 6459, p = 0.19
Left surface area	β = 1216, p = 0.78	β = 191, p = 0.97

Analyses are adjusted for ICV, age_2, sex, age*sex interaction, brain parameter at SHIP-2 and follow-up time, volume refers to GMV.

**Table 5:** Results for brain-gene interactions between PRS for BMI and brain parameters on BMI in SHIP-2.

Predictor*PRS	Coefficient	P-value	95% confidence interval
**Whole brain**			
Right cortical thickness	β = 1151	0.21	[−633, 2934]
Left cortical thickness	β = 644	0.44	[−1006, 2294]
Right volume	β = −0.001	0.77	[−0.009, 0.007]
Left volume	β = −0.002	0.59	[−0.01, 0.006]
Right surface area	β = −0.01	0.21	[−0.04, 0.008]
Left surface area	β = −0.01	0.26	[−0.03, 0.01]
**Orbifrontal cortex**			
Right cortical thickness	β = 363	0.60	[−982, 1707]
Left cortical thickness	β = 191	0.77	[−1084, 1467]
Right volume	β = −0.02	0.69	[−0.14, 0.10]
Left volume	β = −0.01	0.84	[−0.13, 0.11]
Right surface area	β = −0.14	0.41	[−0.46, 0.19]
Left surface area	β = −0.012	0.52	[−0.49, 0.25]
**Anterior cingulate and medial prefrontal cortex**			
Right cortical thickness	β = 575	0.32	[−567, 1718]
Left cortical thickness	β = 79	0.89	[−1078, 1235]
Right volume	β = −0.03	0.53	[−0.14, 0.07]
Left volume	β = −0.01	0.88	[−0.13, 0.12]
Right surface area	β = −0.30	0.056	[−0.61, 0.008]
Left surface area	β = −0.16	0.39	[−0.51, 0.20]

Analyses are adjusted for ICV, age_2, sex and age*sex interaction, volume refers to GMV.

## Discussion

In the present study we aimed at replicating and extending previous findings on associations between high BMI, polygenic risk for elevated BMI, and measures of global as well as regional brain atrophy patterns using a cross-sectional as well as longitudinal approach.

Firstly, our findings support previously described abnormalities of gray matter in areas such as the orbitofrontal (OFC) and the anterior cingulate and medial prefrontal cortex (AC-MPFC) in association with high BMI levels. Although lower global gray matter volume has consistently been observed in individuals with high BMI compared to non-obese control individuals, there has been some controversy on the specific brain regions affected by obesity. In this study we focused on regions of interest (ROIs), which were recently identified in meta-analytic studies^12,13^, which are functionally closely interconnected and which are linked to eating behavior. Specifically, the OFC is related to impulse control^33^, reward^34^, food visual^35^ as well as taste^36^ cues. Negative correlations of obesity with volumes of the AC-MPFC in our study are in line with findings showing reduced volumes and cortical thinning of this region in individuals with childhood obesity^33^ as well as adulthood obesity^37^. The AC-MPFC is a functionally versatile region with a key role in emotion processing and projections in various brain regions involved in cognitive, sensory and motor processes^38^.

Given the key role of the OFC as well as the MPFC for various cognitive domains including learning and memory functioning, decision making^39^ and social cognition^40^, our results lend further support to the concept of obesity as a condition associated with negative impact on brain structure and function including brain regions central to both emotional regulation and cognition. The frequency of reproducibility of these regionally specific results suggest a significant link between these particular regions and obesity.

Secondly, we tested the effect of brain parameters on BMI in a longitudinal setting. We were unable to confirm a significant effect of brain parameters on BMI changes between SHIP-2 and SHIP-3. An individualized prediction of future BMI values based on single brain parameters is not possible according to our study. However, risk loci identified by GWAS were not taken into consideration when utilizing GMV as a prediction tool^41,42^. These findings are in contrast to the results by Opel et al. in 2017^14^, who found that an individualized forecast of the BMI could be made by examining differences in the volume of gray matter. While we applied a similar statistical approach in order to enable a sufficient comparability, the longitudinal approach represents an important difference.

In accordance with this finding our analyses of the genetic score for BMI on longitudinal brain changes in SHIP revealed no significant associations with volumetric brain abnormalities. This calls a direct genetic impact of the risk for obesity on brain structure into question. In the context of the previously reported findings, these results support the concept that obesity and related lifestyle factors may directly impact on patterns of brain structure. Also, it is conceivable that obesity and related factors influence epigenetic regulation of brain metabolism and may thereby impact on the brain structure^43–45^.

In a last step, we tested the cross-sectional interaction between the genetic score of BMI and brain parameters on measured BMI in SHIP-2. Our data demonstrated no interaction between brain parameters and genetic score for BMI on measured BMI in SHIP-2. In particular, we were not able to replicate previous findings by Opel et al.^14^.

Differences may be grounded in different demographic backgrounds of the cohort such as different age and gender distributions within the examined group of individuals. Furthermore, comorbidities within each individual could lead to varying results by possibly masking the effect of PRS for obesity on the brain. Another potential source of variance was the use of clinician-rated somatometric characteristics, while previous studies were mainly based on self-report data.

### Pathophysiological model

Our data indicates structural changes in mean cortical thickness and brain volume of the OFC and the AC-MPFC in participants with elevated BMI cross-sectionally as well as longitudinally, with brain atrophy not being associated with BMI-related predisposition. These findings support a connection between BMI and structural brain alterations. However, based on our data, direct genetic influence for elevated BMI values and consecutive brain alterations is uncertain which promotes the idea of a direct impact of BMI values on structural brain changes.

Previous studies not only showed negative associations between overweight/obesity and brain parameters: for example, different studies on anorexia nervosaan illness characterized with reduced intake of food, revealed signs of brain atrophy^46,47^ and lack of gyrification^48^. When given the right amount of nutrition and regaining weight, the brain was able to recover and brain atrophy was reduced^49^ which makes a connection between lifestyle-related, out-of-the-norm BMI variance and changes in brain morphometry possible. A study with obese patients undergoing bariatric surgery showed similar effects: By losing weight and reducing body fat, gray and white matter integrity was restored^50–54^ making a mediation effect of nutrition in the association between brain atrophy and BMI possible.

S, chronic obesity is not only associated with volumetric decrease in brain mass, it can also lead to cognitive impairment. A meta-analyses by Robinson et al.^55^ showed evidence that mental disorders such as anxiety and depression are associated with elevated BMI values in children and adults^55^. Also highly suggestive evidence for cognitive impairment concerning areas such as reward-related decision making, impulsivity and impairment of overall executive function were found^55^. Benito-Lèon et al. demonstrated that obese participants performed worse in tests quantifying verbal ability, memory function and psychomotoric reaction rate than their normal-/overweight peers^56^ linking obesity to cognitive decline.

However, possible pathways why obesity affects brain function are not fully understood yet. Available data points towards inflammation and vascular dysfunction.

Morys et al. examined the relationship between adiposity and cognitive dysfunction and found that anthropometrics such as BMI levels, waist-to-hip-ratio and body fat percentage are related to increased C-reactive protein levels, hypertension and diabetes^57^.

Janowitz et al.^58^ also demonstrated a link between inflammatory markers and brain atrophy in patients with Alzheimer’s disease. Furthermore, they showed that vascular risk factors such as diabetes, smoking and high triglyceride concentrations which of whom are also found in many obese individuals, are often associated with increased white blood cell counts, suggesting that inflammation is a possible cause of brain atrophy^58^.

### Limitations

Potential selection biases due to exclusion criteria for MRI examinations and individuals with claustrophobia who refused participation may result in unrepresentative samples. Also, underweight participants were not excluded from the sample leading to potential interpretation bias, assuming BMI acts reversely linear to brain volume.

Due to the longitudinal design of this study non-participation of participants at both points of measurement could have induced another potential selection bias.

SHIP only included Caucasians reducing the generalizability of the study but underlining the potential insignificance of genetic effects.

The relatively short time period between both examination points and the moderate sample size may influence the validity of the study due to lower statistical power, which may particularly explain non-significant results of the interaction analysis. Additionally, possible reasoning behind these null results, especially those concerning genetics, could lay in the use of an underpowered polygenic risk score since we are not using the latest and most large-scale one. A highly-powered polygenic score study or a twin study is needed for further examination. Also, functional, cognitive, and affective data was not included as well.

To our knowledge this is the first study to examine volumetric brain differences in a longitudinal setting while observing long-term effects on gray matter in obese participants with PRSs.

Our study provides a significant confirmation documenting the structural differences of gray matter and formulating a possible conclusion between high BMI levels and brain atrophy.

Our study opted for a regions of interest analysis combined with whole brain analysis to conduct a more holistic study. Age bias was prevented by adjusting for age. Measurement errors were minimized by using standardized somatometric examinations. Furthermore, our study was conducted by ascertaining data with the same system (same protocol, same MRI scanner) at both measurement points providing a standardized testing. Participants with metabolic problems such as type II diabetes were not excluded to better naturalistic observation. Nevertheless, other confounding factors influencing the outcome cannot be ruled out.

## Conclusion

Our results provide first evidence for a potential causal relationship between obesity and brain alterations. We found that mainly mean cortical thickness and volume of the OFC and AC-MPFC are negatively affected by higher baseline BMI. However, there is no evidence in our data supporting an association between BMI-related genetic predisposition and brain atrophy. Several mechanisms could play a probable role in the development of brain atrophy in obese individuals: dysregulation of the hypothalamic–pituitary–adrenal axis, inflammation, impaired neuronal plasticity, epigenetic influence and hormonal dysfunction.

## Supplementary Information

Supplementary Information.

## Data Availability

The program that was used for cortical reconstruction of this study is openly available at FreeSurfer (FreeSurfer Version 6, 20127) at: http://surfer.nmr.mgh.harvard.edu. The imputation of genotypes was realized by using Impute2. The software is openly available at: http://mathgen.stats.ox.ac.uk/impute/impute_v2.2.2.html. The brain atlas the findings of this study were based on is available in the supplementary material of Glasser et al. 2016 at Nature: 536:171–178. https://doi.org/10.1038/nature18933. SNP information including *p* values and betas were obtained by using PLINK available at the American Journal of Human Genetics: 81:559–575. https://doi.org/10.1086/519795.

## Code availability

STATA Code for models 1–4 is available in the supplement material.

## References

[1] WHO. Fact Sheets Overweight and Obesity. https://www.who.int/news-room/fact-sheets/detail/obesity-and-overweight. Accessed 12 June 2021 (2021).

[2] Albuquerque D, Nóbrega C, Manco L, Padez C (2017). The contribution of genetics and environment to obesity. Br. Med. Bull..

[3] Locke AE (2015). Genetic studies of body mass index yield new insights for obesity biology. Nature.

[4] Yengo L (2018). Meta-analysis of genome-wide association studies for height and body mass index in ∼700000 individuals of European ancestry. Hum. Mol. Genet..

[5] Koliaki C, Liatis S, Kokkinos A (2019). Obesity and cardiovascular disease: Revisiting an old relationship. Metabolism.

[6] Janowitz D (2015). Association between waist circumference and gray matter volume in 2344 individuals from two adult community-based samples. Neuroimage.

[7] Hamer M, Batty GD (2019). Association of body mass index and waist-to-hip ratio with brain structure: UK Biobank study. Neurology.

[8] Gurholt TP (2021). Population-based body-brain mapping links brain morphology with anthropometrics and body composition. Transl. Psychiatry.

[9] Pannacciulli N (2006). Brain abnormalities in human obesity: A voxel-based morphometric study. Neuroimage.

[10] Vainik U (2018). Neurobehavioral correlates of obesity are largely heritable. Proc. Natl. Acad. Sci. U. S. A..

[11] Alonso-Alonso M, Pascual-Leone A (2007). The right brain hypothesis for obesity. JAMA.

[12] Chen EY, Eickhoff SB, Giovannetti T, Smith DV (2020). Obesity is associated with reduced orbitofrontal cortex volume: A coordinate-based meta-analysis. NeuroImage Clin..

[13] García-García I (2019). Neuroanatomical differences in obesity: Meta-analytic findings and their validation in an independent dataset. Int. J. Obes..

[14] Opel N (2017). Prefrontal gray matter volume mediates genetic risks for obesity. Mol. Psychiatry.

[15] Opel N (2020). Brain structural abnormalities in obesity: Relation to age, genetic risk, and common psychiatric disorders: Evidence through univariate and multivariate mega-analysis including 6420 participants from the ENIGMA MDD working group. Mol. Psychiatry.

[16] Winkler AM (2010). Cortical thickness or grey matter volume? The importance of selecting the phenotype for imaging genetics studies. Neuroimage.

[17] Bruce-Keller, A. J., Keller, J. N. & Morrison, C. D. Obesity and vulnerability of the CNS. *Biochim. Biophys. Acta BBA Mol. Basis Dis.* **1792**, 395–400 (2009). 10.1016/j.bbadis.2008.10.004 PMC2699212 18992327

[18] Völzke H (2011). Cohort profile: The study of health in Pomerania. Int. J. Epidemiol..

[19] Hegenscheid K (2009). Whole-body magnetic resonance imaging of healthy volunteers: Pilot study results from the population-based SHIP study. ROFO. Fortschr. Geb. Rontgenstr. Nuklearmed..

[20] Grabe HJ (2014). Alexithymia and brain gray matter volumes in a general population sample. Hum. Brain Mapp..

[21] Buckner RL (2004). A unified approach for morphometric and functional data analysis in young, old, and demented adults using automated atlas-based head size normalization: reliability and validation against manual measurement of total intracranial volume. Neuroimage.

[22] Sled JG, Zijdenbos AP, Evans AC (1998). A nonparametric method for automatic correction of intensity nonuniformity in MRI data. IEEE Trans. Med. Imaging.

[23] Fischl B, Liu A, Dale AM (2001). Automated manifold surgery: Constructing geometrically accurate and topologically correct models of the human cerebral cortex. IEEE Trans. Med. Imaging.

[24] Ségonne F, Pacheco J, Fischl B (2007). Geometrically accurate topology-correction of cortical surfaces using nonseparating loops. IEEE Trans. Med. Imaging.

[25] Dale AM, Fischl B, Sereno MI (1999). Cortical surface-based analysis I. Segmentation and surface reconstruction. Neuroimage.

[26] Dale AM, Sereno MI (1993). Improved localizadon of cortical activity by combining EEG and MEG with MRI cortical surface reconstruction: A linear approach. J. Cogn. Neurosci..

[27] Fischl B, Dale AM (2000). Measuring the thickness of the human cerebral cortex from magnetic resonance images. Proc. Natl. Acad. Sci. U. S. A..

[28] Glasser MF (2016). A multi-modal parcellation of human cerebral cortex. Nature.

[29] Kringelbach ML (2005). The human orbitofrontal cortex: Linking reward to hedonic experience. Nat. Rev. Neurosci..

[30] Teumer A (2019). Genome-wide association meta-analyses and fine-mapping elucidate pathways influencing albuminuria. Nat. Commun..

[31] Purcell S (2007). PLINK: A tool set for whole-genome association and population-based linkage analyses. Am. J. Hum. Genet..

[32] Harrell FE (2001). Regression Modeling Strategies with Applications to Linear Models, Logistic Regression, and Survival Analysis.

[33] Marqués-Iturria I (2013). Frontal cortical thinning and subcortical volume reductions in early adulthood obesity. Psychiatry Res..

[34] Oldham S (2018). The anticipation and outcome phases of reward and loss processing: A neuroimaging meta-analysis of the monetary incentive delay task. Hum. Brain Mapp..

[35] van der Laan LN, de Ridder DTD, Viergever MA, Smeets PAM (2011). The first taste is always with the eyes: A meta-analysis on the neural correlates of processing visual food cues. Neuroimage.

[36] Veldhuizen MG (2011). Identification of human gustatory cortex by activation likelihood estimation. Hum. Brain Mapp..

[37] Medic N (2016). Increased body mass index is associated with specific regional alterations in brain structure. Int. J. Obes..

[38] Etkin A, Egner T, Kalisch R (2011). Emotional processing in anterior cingulate and medial prefrontal cortex. Trends Cogn. Sci..

[39] Wikenheiser AM, Schoenbaum G (2016). Over the river, through the woods: Cognitive maps in the hippocampus and orbitofrontal cortex. Nat. Rev. Neurosci..

[40] Kong F (2019). Neural correlates of social well-being: Gray matter density in the orbitofrontal cortex predicts social well-being in emerging adulthood. Soc. Cogn. Affect. Neurosci..

[41] Hung C-F (2015). A genetic risk score combining 32 SNPs is associated with body mass index and improves obesity prediction in people with major depressive disorder. BMC Med..

[42] Lee JS, Cheong HS, Shin H-D (2017). BMI prediction within a Korean population. PeerJ.

[43] van Dijk SJ (2015). Epigenetics and human obesity. Int. J. Obes..

[44] van Dijk SJ, Tellam RL, Morrison JL, Muhlhausler BS, Molloy PL (2015). Recent developments on the role of epigenetics in obesity and metabolic disease. Clin. Epigenet..

[45] Lopomo A, Burgio E, Migliore L (2016). Epigenetics of obesity. Prog. Mol. Biol. Transl. Sci..

[46] Titova OE, Hjorth OC, Schiöth HB, Brooks SJ (2013). Anorexia nervosa is linked to reduced brain structure in reward and somatosensory regions: A meta-analysis of VBM studies. BMC Psychiatry.

[47] Bernardoni F (2018). Nutritional status affects cortical folding: Lessons learned from anorexia nervosa. Biol. Psychiatry.

[48] Bernardoni F (2016). Weight restoration therapy rapidly reverses cortical thinning in anorexia nervosa: A longitudinal study. Neuroimage.

[49] King JA (2015). Global cortical thinning in acute anorexia nervosa normalizes following long-term weight restoration. Biol. Psychiatry.

[50] Nota MHC (2020). Obesity affects brain structure and function- rescue by bariatric surgery?. Neurosci. Biobehav. Rev..

[51] Bohon C, Geliebter A (2019). Change in brain volume and cortical thickness after behavioral and surgical weight loss intervention. NeuroImage Clin..

[52] Bohon C, Garcia LC, Morton JM (2018). Changes in cerebral cortical thickness related to weight loss following bariatric surgery. Obes. Surg..

[53] Rullmann M (2018). Gastric-bypass surgery induced widespread neural plasticity of the obese human brain. Neuroimage.

[54] Tuulari JJ (2016). Bariatric surgery induces white and grey matter density recovery in the morbidly obese: A voxel-based morphometric study. Hum. Brain Mapp..

[55] Robinson E, Roberts C, Vainik U, Jones A (2020). The psychology of obesity: An umbrella review and evidence-based map of the psychological correlates of heavier body weight. Neurosci. Biobehav. Rev..

[56] Benito-León J, Mitchell AJ, Hernández-Gallego J, Bermejo-Pareja F (2013). Obesity and impaired cognitive functioning in the elderly: A population-based cross-sectional study (NEDICES). Eur. J. Neurol..

[57] Morys F, Dadar M, Dagher A (2021). Association between midlife obesity and its metabolic consequences, cerebrovascular disease, and cognitive decline. J. Clin. Endocrinol. Metab..

[58] Janowitz D (2019). Inflammatory markers and imaging patterns of advanced brain aging in the general population. Brain Imaging Behav..

