# Supplementary material for: Body mass index but not genetic risk is longitudinally associated with altered structural brain parameters
Source: Sci Rep. 2021 Dec 20;11:24246. doi: 10.1038/s41598-021-03343-3 (PMC8688483; doi:10.1038/s41598-021-03343-3)
Supplement: Supplementary file 1 — Supplementary Information. [file 41598_2021_3343_MOESM1_ESM.docx]

**Supplement**

**1. Prefrontal Cortex Regions**

Glasser et al. ^1^ provided the underlying brain atlas for this study. Based on this atlas, we have defined the two areas that were of interest to us.

The AC-PFC consists of 15 sub-areas: 33pr, p24pr, a24pr, p24, a24, p32pr, a32pr, d32, p32, s32, 8BM, 9m, 10v, 10r, and 25 (see supplementary material of ^1^).

The OFC, referred to as orbital and polar frontal cortex by Glasser et al., is subdivided in 11 areas: 47s, 47m, a47r, 11l, 13l, a10p, p10p, 10pp, 10d, orbitofrontal complex and posterior orbitofrontal complex (see supplementary material of ^1^).

| Parcel abbreviation | Anatomical name |
| --- | --- |
| 33pr | Area 33 prime |
| p24pr | Area posterior 24 prime |
| a24pr | Anterior 24 prime |
| p24 | Area posterior 24 |
| a24 | Area a24 |
| p32pr | Area p32 prime |
| a32pr | Area anterior 32 prime |
| d32 | Area dorsal 32 |
| s32 | Area s32 |
| 8BM | Area 8BM |
| 9m | Area 9 Middle |
| 10v | Area 10v |
| 10r | Area 10r |
| 25 | Area 25 |
| 47s | Area 47s |
| 47m | Area 47m |
| a47r | Area anterior 47r |
| 11l | Area 11l |
| 13l | Area 13l |
| a10p | Area anterior 10p |
| p10p | Area posterior 10p |
| 10pp | Polar 10p |
| 10d | Area 10d |
| OFC | Orbitofrontal complex |
| pOFC | Posterior orbitofrontal complex |

**2. Material and Methods**

**2.1. STATA code**

* variable description for SHIP-2 (s2) and SHIP-3 (s3):

*

* - area_l/r: whole brain area measurement for left and right hemisphere

* - vol_l/r: whole brain volume for left and right hemisphere

* - thick_l/r: whole brain mean thickness for left and right hemisphere

* - ofc_...: analog for orbifrontal cortex (OFC)

* - pfc_...: analog for prefrontal cortex (PFC)

* - bmi: Body Mass Index

* - age_spline1-3: three age splines for nonlinear correction of age

* - sex: sex (1: male, 2: female) on an individual

* - fup_duration: follow-up duration between SHIP-2 and SHIP-3

* - icv: intracranial volume at SHIP-2

* - QC: quality control parameter that includes medical as well as technical quality check

* - prs_bmi_005: polygenic score for BMI (based on Locke et al. 2015 GWAS) with p-value threshold p<0.05

*

********************************************************************************

set pformat %-5.1e

*//Hypothesis 1: BMI predicts brain atrophy

*//Whole Brain measurements

reg s3_area_l c.bmi_s2 s2_area_l c.age_spline1##sex c.age_spline2##sex c.age_spline3##sex fup_duration icv if QC==1, vce(robust)

reg s3_area_r c.bmi_s2 s2_area_r c.age_spline1##sex c.age_spline2##sex c.age_spline3##sex fup_duration icv if QC==1, vce(robust)

reg s3_vol_l c.bmi_s2 s2_vol_l c.age_spline1##sex c.age_spline2##sex c.age_spline3##sex fup_duration icv if QC==1, vce(robust)

reg s3_vol_r c.bmi_s2 s2_vol_r c.age_spline1##sex c.age_spline2##sex c.age_spline3##sex fup_duration icv if QC==1, vce(robust)

reg s3_thick_l c.bmi_s2 s2_thick_l c.age_spline1##sex c.age_spline2##sex c.age_spline3##sex fup_duration icv if QC==1, vce(robust)

reg s3_thick_r c.bmi_s2 s2_thick_r c.age_spline1##sex c.age_spline2##sex c.age_spline3##sex fup_duration icv if QC==1, vce(robust)

*//OFC

reg s3_ofc_area_l c.bmi_s2 s2_ofc_area_l c.age_spline1##sex c.age_spline2##sex c.age_spline3##sex fup_duration icv if QC==1, vce(robust)

reg s3_ofc_area_r c.bmi_s2 s2_ofc_area_r c.age_spline1##sex c.age_spline2##sex c.age_spline3##sex fup_duration icv if QC==1, vce(robust)

reg s3_ofc_vol_l c.bmi_s2 s2_ofc_vol_l c.age_spline1##sex c.age_spline2##sex c.age_spline3##sex fup_duration icv if QC==1, vce(robust)

reg s3_ofc_vol_r c.bmi_s2 s2_ofc_vol_r c.age_spline1##sex c.age_spline2##sex c.age_spline3##sex fup_duration icv if QC==1, vce(robust)

reg s3_ofc_thick_l c.bmi_s2 s2_ofc_thick_l c.age_spline1##sex c.age_spline2##sex c.age_spline3##sex fup_duration icv if QC==1, vce(robust)

reg s3_ofc_thick_r c.bmi_s2 s2_ofc_thick_r c.age_spline1##sex c.age_spline2##sex c.age_spline3##sex fup_duration icv if QC==1, vce(robust)

*//PFC

reg s3_pfc_area_l c.bmi_s2 s2_pfc_area_l c.age_spline1##sex c.age_spline2##sex c.age_spline3##sex fup_duration icv if QC==1, vce(robust)

reg s3_pfc_area_r c.bmi_s2 s2_pfc_area_r c.age_spline1##sex c.age_spline2##sex c.age_spline3##sex fup_duration icv if QC==1, vce(robust)

reg s3_pfc_vol_l c.bmi_s2 s2_pfc_vol_l c.age_spline1##sex c.age_spline2##sex c.age_spline3##sex fup_duration icv if QC==1, vce(robust)

reg s3_pfc_vol_r c.bmi_s2 s2_pfc_vol_r c.age_spline1##sex c.age_spline2##sex c.age_spline3##sex fup_duration icv if QC==1, vce(robust)

reg s3_pfc_thick_l c.bmi_s2 s2_pfc_thick_l c.age_spline1##sex c.age_spline2##sex c.age_spline3##sex fup_duration icv if QC==1, vce(robust)

reg s3_pfc_thick_r c.bmi_s2 s2_pfc_thick_r c.age_spline1##sex c.age_spline2##sex c.age_spline3##sex fup_duration icv if QC==1, vce(robust)

******************************************************************************

******************************************************************************

* Hypothesis 2: PRS for BMI predicts brain atrophy

* without additional adjustment for baseline BMI at SHIP-2

*//Whole Brain

reg s3_area_l c.prs_bmi_005 s2_area_l c.age_spline1##sex c.age_spline2##sex c.age_spline3##sex fup_duration icv if QC==1, vce(robust)

reg s3_area_r c.prs_bmi_005 s2_area_r c.age_spline1##sex c.age_spline2##sex c.age_spline3##sex fup_duration icv if QC==1, vce(robust)

reg s3_vol_l c.prs_bmi_005 s2_vol_l c.age_spline1##sex c.age_spline2##sex c.age_spline3##sex fup_duration icv if QC==1, vce(robust)

reg s3_vol_r c.prs_bmi_005 s2_vol_r c.age_spline1##sex c.age_spline2##sex c.age_spline3##sex fup_duration icv if QC==1, vce(robust)

reg s3_thick_l c.prs_bmi_005 s2_thick_l c.age_spline1##sex c.age_spline2##sex c.age_spline3##sex fup_duration icv if QC==1, vce(robust)

reg s3_thick_r c.prs_bmi_005 s2_thick_r c.age_spline1##sex c.age_spline2##sex c.age_spline3##sex fup_duration icv if QC==1, vce(robust)

*//OFC

reg s3_ofc_area_l c.prs_bmi_005 s2_ofc_area_l c.age_spline1##sex c.age_spline2##sex c.age_spline3##sex fup_duration icv if QC==1, vce(robust)

reg s3_ofc_area_r c.prs_bmi_005 s2_ofc_area_r c.age_spline1##sex c.age_spline2##sex c.age_spline3##sex fup_duration icv if QC==1, vce(robust)

reg s3_ofc_vol_l c.prs_bmi_005 s2_ofc_vol_l c.age_spline1##sex c.age_spline2##sex c.age_spline3##sex fup_duration icv if QC==1, vce(robust)

reg s3_ofc_vol_r c.prs_bmi_005 s2_ofc_vol_r c.age_spline1##sex c.age_spline2##sex c.age_spline3##sex fup_duration icv if QC==1, vce(robust)

reg s3_ofc_thick_l c.prs_bmi_005 s2_ofc_thick_l c.age_spline1##sex c.age_spline2##sex c.age_spline3##sex fup_duration icv if QC==1, vce(robust)

reg s3_ofc_thick_r c.prs_bmi_005 s2_ofc_thick_r c.age_spline1##sex c.age_spline2##sex c.age_spline3##sex fup_duration icv if QC==1, vce(robust)

*//PFC

reg s3_pfc_area_l c.prs_bmi_005 s2_pfc_area_l c.age_spline1##sex c.age_spline2##sex c.age_spline3##sex fup_duration icv if QC==1, vce(robust)

reg s3_pfc_area_r c.prs_bmi_005 s2_pfc_area_r c.age_spline1##sex c.age_spline2##sex c.age_spline3##sex fup_duration icv if QC==1, vce(robust)

reg s3_pfc_vol_l c.prs_bmi_005 s2_pfc_vol_l c.age_spline1##sex c.age_spline2##sex c.age_spline3##sex fup_duration icv if QC==1, vce(robust)

reg s3_pfc_vol_r c.prs_bmi_005 s2_pfc_vol_r c.age_spline1##sex c.age_spline2##sex c.age_spline3##sex fup_duration icv if QC==1, vce(robust)

reg s3_pfc_thick_l c.prs_bmi_005 s2_pfc_thick_l c.age_spline1##sex c.age_spline2##sex c.age_spline3##sex fup_duration icv if QC==1, vce(robust)

reg s3_pfc_thick_r c.prs_bmi_005 s2_pfc_thick_r c.age_spline1##sex c.age_spline2##sex c.age_spline3##sex fup_duration icv if QC==1, vce(robust)

* with additional adjustment for baseline BMI at SHIP-2

*//Whole Brain

reg s3_area_l c.prs_bmi_005 c.bmi_s2 s2_area_l c.age_spline1##sex c.age_spline2##sex c.age_spline3##sex fup_duration icv if QC==1, vce(robust)

reg s3_area_r c.prs_bmi_005 c.bmi_s2 s2_area_r c.age_spline1##sex c.age_spline2##sex c.age_spline3##sex fup_duration icv if QC==1, vce(robust)

reg s3_vol_l c.prs_bmi_005 c.bmi_s2 s2_vol_l c.age_spline1##sex c.age_spline2##sex c.age_spline3##sex fup_duration icv if QC==1, vce(robust)

reg s3_vol_r c.prs_bmi_005 c.bmi_s2 s2_vol_r c.age_spline1##sex c.age_spline2##sex c.age_spline3##sex fup_duration icv if QC==1, vce(robust)

reg s3_thick_l c.prs_bmi_005 c.bmi_s2 s2_thick_l c.age_spline1##sex c.age_spline2##sex c.age_spline3##sex fup_duration icv if QC==1, vce(robust)

reg s3_thick_r c.prs_bmi_005 c.bmi_s2 s2_thick_r c.age_spline1##sex c.age_spline2##sex c.age_spline3##sex fup_duration icv if QC==1, vce(robust)

*//OFC

reg s3_ofc_area_l c.prs_bmi_005 c.bmi_s2 s2_ofc_area_l c.age_spline1##sex c.age_spline2##sex c.age_spline3##sex fup_duration icv if QC==1, vce(robust)

reg s3_ofc_area_r c.prs_bmi_005 c.bmi_s2 s2_ofc_area_r c.age_spline1##sex c.age_spline2##sex c.age_spline3##sex fup_duration icv if QC==1, vce(robust)

reg s3_ofc_vol_l c.prs_bmi_005 c.bmi_s2 s2_ofc_vol_l c.age_spline1##sex c.age_spline2##sex c.age_spline3##sex fup_duration icv if QC==1, vce(robust)

reg s3_ofc_vol_r c.prs_bmi_005 c.bmi_s2 s2_ofc_vol_r c.age_spline1##sex c.age_spline2##sex c.age_spline3##sex fup_duration icv if QC==1, vce(robust)

reg s3_ofc_thick_l c.prs_bmi_005 c.bmi_s2 s2_ofc_thick_l c.age_spline1##sex c.age_spline2##sex c.age_spline3##sex fup_duration icv if QC==1, vce(robust)

reg s3_ofc_thick_r c.prs_bmi_005 c.bmi_s2 s2_ofc_thick_r c.age_spline1##sex c.age_spline2##sex c.age_spline3##sex fup_duration icv if QC==1, vce(robust)

*//PFC

reg s3_pfc_area_l c.prs_bmi_005 c.bmi_s2 s2_pfc_area_l c.age_spline1##sex c.age_spline2##sex c.age_spline3##sex fup_duration icv if QC==1, vce(robust)

reg s3_pfc_area_r c.prs_bmi_005 c.bmi_s2 s2_pfc_area_r c.age_spline1##sex c.age_spline2##sex c.age_spline3##sex fup_duration icv if QC==1, vce(robust)

reg s3_pfc_vol_l c.prs_bmi_005 c.bmi_s2 s2_pfc_vol_l c.age_spline1##sex c.age_spline2##sex c.age_spline3##sex fup_duration icv if QC==1, vce(robust)

reg s3_pfc_vol_r c.prs_bmi_005 c.bmi_s2 s2_pfc_vol_r c.age_spline1##sex c.age_spline2##sex c.age_spline3##sex fup_duration icv if QC==1, vce(robust)

reg s3_pfc_thick_l c.prs_bmi_005 c.bmi_s2 s2_pfc_thick_l c.age_spline1##sex c.age_spline2##sex c.age_spline3##sex fup_duration icv if QC==1, vce(robust)

reg s3_pfc_thick_r c.prs_bmi_005 c.bmi_s2 s2_pfc_thick_r c.age_spline1##sex c.age_spline2##sex c.age_spline3##sex fup_duration icv if QC==1, vce(robust)

*****************************************************************************

*****************************************************************************

* Hypothesis 3: brain mesurement at SHIP-2 predicts BMI changes

* whole brain

foreach j of varlist s2_area_l s2_area_r s2_vol_l s2_vol_r s2_thick_l s2_thick_r{

reg bmi_s3 `j' c.age_spline1##sex c.age_spline2##sex c.age_spline3##sex fup_duration c.bmi_s2 icv if QC==1, vce(robust)

}

* OFC

foreach j of varlist s2_ofc_area_l s2_ofc_area_r s2_ofc_vol_l s2_ofc_vol_r s2_ofc_thick_l s2_ofc_thick_r{

reg bmi_s3 `j' c.age_spline1##sex c.age_spline2##sex c.age_spline3##sex fup_duration c.bmi_s2 icv if QC==1, vce(robust)

}

* PFC

foreach j of varlist s2_pfc_area_l s2_pfc_area_r s2_pfc_vol_l s2_pfc_vol_r s2_pfc_thick_l s2_pfc_thick_r{

reg bmi_s3 `j' c.age_spline1##sex c.age_spline2##sex c.age_spline3##sex fup_duration c.bmi_s2 icv if QC==1, vce(robust)

}

*****************************************************************************

*****************************************************************************

* Hypothesis 4: PRS for BMI and brain volume exhibit an interaction effect on BMI in SHIP-2 (cross-sectional setting)

* whole brain

foreach j of varlist s2_area_l s2_area_r s2_vol_l s2_vol_r s2_thick_l s2_thick_r{

bmi_s2 c.`j'##c.prs_bmi_005 c.age_spline1##sex c.age_spline2##sex c.age_spline3##sex icv if QC==1, vce(robust)

}

* OFC

foreach j of varlist s2_ofc_area_l s2_ofc_area_r s2_ofc_vol_l s2_ofc_vol_r s2_ofc_thick_l s2_ofc_thick_r{

bmi_s2 c.`j'##c.prs_bmi_005 c.age_spline1##sex c.age_spline2##sex c.age_spline3##sex icv if QC==1, vce(robust)

}

* PFC

foreach j of varlist s2_pfc_area_l s2_pfc_area_r s2_pfc_vol_l s2_pfc_vol_r s2_pfc_thick_l s2_pfc_thick_r{

bmi_s2 c.`j'##c.prs_bmi_005 c.age_spline1##sex c.age_spline2##sex c.age_spline3##sex icv if QC==1, vce(robust)

}

**2. Results**

Table Reviewer S. Results for cross-sectional effects of BMI at SHIP-2 on brain parameters at SHIP-2 and additionally cross-sectional effects of BMI at SHIP-3 on brain parameters at SHIP-3

|  |  | SHIP-2 |  |  | SHIP-3 |  |
| --- | --- | --- | --- | --- | --- | --- |
| Outcome | Coefficient | P-value | 95% Confidence Interval | Coefficient | P-value | 95% CI |
| Whole brain |  |  |  |  |  |  |
| Right cortical thickness | **β=-0.003** | **0.008** | **[-0.004, -0.0007]** | **-0.003** | **0.006** | **[-0.005, -0.0008]** |
| Left cortical thickness | β=-0.002 | 0.020 | [-0.004, -0.0003] | **-0.0025** | **0.008** | **[-0.004, -0.0007]** |
| Right volume | β=-237.75 | 0.021 | [-440.24, -35.27] | -205.39 | 0.039 | [-400.79, -10.00] |
| Left volume | **β=-255.18** | **0.013** | **[-456.26, -54.11]** | -218.58 | 0.023 | [-406.32, -30.84] |
| Right surface area | β=25.75 | 0.57 | [-62.41, 113.92] | 49.89 | 0.23 | [-31.13, 130.91] |
| Left surface area | β=10.15 | 0.81 | [-74.49, 94.79] | 44.61 | 0.26 | [-33.56, 122.78] |
|  |  |  |  |  |  |  |
| Orbitofrontal Cortex | | | |  |  |  |
| Right cortical thickness | β=-0.0013 | 0.38 | [-0.004, 0.0016] | -0.004 | 0.009 | [-0.007, -0.001] |
| Left cortical thickness | β=-0.003 | 0.046 | [-0.006, -0.0001] | -0.005 | 0.001 | [-0.008, -0.002] |
| Right volume | β=-23.19 | 0.037 | [-44.98, -1.41] | -32.83 | 0.002 | [-53.39, -12.27] |
| Left volume | **β=-26.89** | **0.014** | **[-48.32, -5.46]** | **-35.32** | **0.001** | **[-56.04, -14.60]** |
| Right surface area | β=-2.38 | 0.51 | [-9.41, 4.64] | 1.09 | 0.75 | [-5.72, 7.90] |
| Left surface area | β=-0.73 | 0.85 | [-8.15, 6.69] | 1.82 | 0.62 | [-5.37, 9.00] |
|  |  |  |  |  |  |  |
| Anterior cingulate and medial prefrontal Cortex | | |  |  |  |  |
| Right cortical thickness | **β=-0.006** | **0.001** | **[-0.009, -0.003]** | **-0.007** | **1.7E-5** | **[-0.01, -0.004]** |
| Left cortical thickness | **β=-0.005** | **0.0001** | **[-0.008, -0.003]** | **-0.006** | **6.5E-5** | **[-0.009, -0.003]** |
| Right volume | **β=-28.47** | **0.015** | **[-51.34, -5.59]** | **-30.20** | **0.01** | **[-53.15, -7.24]** |
| Left volume | β=-28.34 | 0.019 | [-52.06, -4.61] | -21.86 | 0.035 | [-42.22, -1.49] |
| Right surface area | β=4.21 | 0.29 | [-3.55, 11.97] | 4.72 | 0.19 | [-2.29, 11.72] |
| Left surface area | β=2.41 | 0.52 | [-4.87, 9.68] | 4.06 | 0.21 | [-2.30, 10.43] |

^significant results are highlighted in bold (p<0.017); analyses are adjusted for ICV_2/3, age_2/3, sex, age_2/3*sex interaction, brain parameter at SHIP2/3^

**2. References**

1. Glasser, M. F. *et al.* A multi-modal parcellation of human cerebral cortex. *Nature* **536**, 171–178 (2016).
